# Disordered Nanohole Patterns in Metal-Insulator Multilayer for Ultra-broadband Light Absorption: Atomic Layer Deposition for Lithography Free Highly repeatable Large Scale Multilayer Growth

**DOI:** 10.1038/s41598-017-15312-w

**Published:** 2017-11-08

**Authors:** Amir Ghobadi, Hodjat Hajian, Sina Abedini Dereshgi, Berkay Bozok, Bayram Butun, Ekmel Ozbay

**Affiliations:** 1 NANOTAM-Nanotechnology Research Center, Bilkent University, 06800 Ankara, Turkey; 2 Department of Electrical and Electronics Engineering, Bilkent University, 06800 Ankara, Turkey; 3 Department of Physics, Bilkent University, 06800 Ankara, Turkey; 4 UNAM-Institute of Materials Science and Nanotechnology, Bilkent University, Ankara, Turkey

## Abstract

In this paper, we demonstrate a facile, lithography free, and large scale compatible fabrication route to synthesize an ultra-broadband wide angle perfect absorber based on metal-insulator-metal-insulator (MIMI) stack design. We first conduct a simulation and theoretical modeling approach to study the impact of different geometries in overall stack absorption. Then, a Pt-Al_2_O_3_ multilayer is fabricated using a single atomic layer deposition (ALD) step that offers high repeatability and simplicity in the fabrication step. In the best case, we get an absorption bandwidth (BW) of 600 nm covering a range of 400 nm–1000 nm. A substantial improvement in the absorption BW is attained by incorporating a plasmonic design into the middle Pt layer. Our characterization results demonstrate that the best configuration can have absorption over 0.9 covering a wavelength span of 400 nm–1490 nm with a BW that is 1.8 times broader compared to that of planar design. On the other side, the proposed structure retains its absorption high at angles as wide as 70°. The results presented here can serve as a beacon for future performance enhanced multilayer designs where a simple fabrication step can boost the overall device response without changing its overall thickness and fabrication simplicity.

## Introduction

Light manipulation in subwavelength geometries is an attribute that nanophotonics can offer. Propagation and guiding, beaming, and confinement of the light are the most studied concepts in this area. The design of an ideal “black body” absorber with the absorption of unity is one of the ultimate goals in the field of subwavelength light confinement. Black absorbers are required in a variety of applications spanning from thermal imaging^1–3^, to photovoltaics^4^. An ideal perfect absorber should have certain characteristics, including (1) broadband absorption, (2) polarization insensitivity, (3) omnidirectionality, and (4) compatibility with large scale fabrication methods. Up to now, several methods and architectures are exploited to obtain the above figures of merit of a black absorber. One of the most commonly used approaches is to utilize metal-insulator-metal (MIM) multilayer stack to confine the light in the subwavelength cavity. In general, in this cavity design, the bottom metal acts as a mirror that reflects light in the entire range of wavelengths. On the other side, the top metal layer is patterned into nanoscale resonant units that confine light into the cavity. Various nano patches have been employed to form these black absorbers. In one of the pioneer works, Aydin introduced a top metal design of crossed trapezoidal arrays that absorb light with an average amount above 70% across the entire visible frequency (400 nm–700 nm)^5^. Later, several other geometries were assigned to the top layer pattern to provide a substantial boost in MIM absorption capability. Nano disc (with the same and different radii)^6–8^, nano square^9–11^, nano ring^12^, multi shape nanostructures^13–15^, ultra-sharp convex grooves and sharp triangular shapes^16^, and other innovative designs^17,18^ are examples of these proposals. The main idea in these designs are exciting adjacent resonances using differently sized nano resonators where the superposition of these narrow resonances will result in a broad response; however, this breadth is achieved at the expense of reduced absorption. Other common geometries are tapered nano gratings and nano pyramids where pyramids are composed of double or multilayer metal-insulator stacks^19–22^. This architecture provides a gradual matching between air and substrate impedances and ensures a broad light absorption. Although some of these absorbers propose good optical performance, they all suffer from fabrication complexity. The use of E-beam lithography (EBL), to pattern the top layer, is an obstacle that restricts their compatibility for large scale fabrication. Recently, some efforts have been devoted to surmount this barrier. Employing a lossy top metallic layer with a thickness of a couple of nanometers was one of the applied approaches to get an EBL free black absorber. It was proved that an asymmetric Cr-SiO_2_-Cr MIM stack, where the top layer is just 3 nm thick, can absorb light with an amount near unity over 450 nm–800 nm^23^.

A substantial improvement can be added up to this design by using metal-insulator-metal-insulator (MIMI) or multi metal-insulator (MI) pair configurations. In one of the first studies, it was theoretically demonstrated that the use of periodic MI pairs can provide impedance matching in a broad frequency range^24^. Several different metal and insulators are utilized to get ultra-broadband light absorption from these multilayer designs. W-Al_2_O_3_^25^, Cr-SiO_2_^26^, W-SiO_2_^26^, Mo-SiO_2_^26^, Ag-Si^27^, and Ni-SiO_2_^28^ are some examples of these MI pairs to obtain perfect broadband absorption. It was theoretically and experimentally proved that the Cr-SiO_2_ multilayer has the best impedance matching and, consequently, it can provide light absorption over 90% in a broad wavelength range between 400 nm–1400 nm^26^. Different from MIM design that needs EBL to fabricate plasmonic top patch layer, MIMI planar design does not involve any EBL process and its bandwidth can be extended toward near-infrared (NIR) regime by increasing the number of MI pairs. In these stacks, essentially, an ultrathin metallic coating is sandwiched between two insulator layers. These metal layers are coated via physical vapor deposition (PVD) systems. However, PVD methods have less control on coating morphology compared to other techniques, such as chemical vapor deposition (CVD), especially for ultrathin metal layers. A multi-step fabrication route comes with another limiting factor that impedes their reproduction capacity. Apart from fabrication point of view, these designs do not benefit from the light confinement in plasmonic nano units due to their planar morphology. Considering the fact that these designs are based on the field-penetration and reflected-wave cancellation principle, the proposed plasmonic geometry should not disturb the inherent response of the device. In other words, an ideal plasmonic structure is obtained by keeping the layer continuous. This can be attained using nanoholes drilled inside middle metal layer. However, the introduction of these nanostructures would force us to utilize the EBL process, which is not desirable in terms of fabrication complexity. In a recent study, we showed that the use of multi-thickness continuous layer in the middle metal layer can enhance light absorption capacity of the multilayer. In this design, every part of the metal gets activated in a specific frequency range in which the overall response (which is superposition of each of these resonant modes) will be broad. The proposed design was obtained using two-step deposition of the Pt layer where the first layer had a nano network morphology obtained through an annealing process, covered with a thin metal layer to achieve multi-thick middle layer^29^.

In this study, we propose an ultrathin, EBL free, ultra-broadband, MIMI multilayer stack, containing plasmonic nanoholes, which is fabricated using an atomic layer deposition (ALD) tool followed with a simple annealing step. Atomic layer deposition is a self-limiting growth process that offers uniform and conformal coating of non-line-of-sight surfaces including high aspect ratio features. This method ensures the uniform and conformal coating of pinhole-free, nanometer-thick layers over the entire substrate surface, which is pivotal for improving material/device performance. This method offers a single step process to obtain multilayer MI stack which makes it an excellent choice for large scale applications. In the current work, using simulations, we first investigate the impact of different geometries on the performance of Pt-Al_2_O_3_-Pt-Al_2_O_3_ stack where the whole structure is fabricated through a single ALD step. Later, to substantially improve the absorption bandwidth, the middle ultrathin planar Pt layer is turned to disordered, widely size distributed nano-holes employing a simple annealing process at 800 °C. To have a better insight into our results, we have also conducted a theoretical study on the differences of planar and nanoholes cases. Our results show that while Pt-Al_2_O_3_ optical behavior is quite close to an ideal metal for impedance matching, this accordance is even better in the case of nanoholes integrated design. Methods and findings of this paper propose a facile approach to design multilayer plasmonic stacks with simple and highly repeatable fabrication routes. Moreover, these methods can be extended to other types of metals and insulators where a broader response can be achieved using the proposed plasmonic scheme without changing the overall thickness of the system.

## Methods

### Synthesis of Metal-insulator Pair by Atomic Layer Deposition

Briefly, first, quartz substrate is cleaned in a sonication bath with acetone, ethanol, and de-ionized (DI) water each for 15 min and then dried by N_2_ flow. Then, a Pt- Al_2_O_3_ multilayer is grown on this substrate using the ALD method. Both Pt and Al_2_O_3_ depositions are carried out at 250 °C in an ALD reactor (Cambridge Nanotech Savannah S100) employing Al(CH_3_)_3_ and MeCpPtMe_3_ solution as deposition precursors, respectively. For the case of a Pt layer, the pulse and purge times are chosen to be 0.1 s and 20 s while for Al_2_O_3_, these amounts are 0.015 s and 10 s. As an oxygen source, water and ozone are utilized for Al_2_O_3_ and Pt, respectively. In the multilayer design, the bottom Pt layer thickness is fixed at 50 nm. The other three layers thicknesses are adjusted by controlling the number of deposition cycles. The growth rate for Pt and Al_2_O_3_ layers is estimated to be 0.93 Å per cycle and 1.08 Å per cycle, respectively.

### Nanohole Formation Using the Dewetting Process

To form nano structures from a thin metal layer, we utilized furnace based annealing. In this method, the sample is put in a tube furnace and the temperature is raised up to 800 °C at a rate of 30 °C/min. Then, it is kept at this temperature for 8 min for dewetting treatment and after that duration the furnace is opened to room temperature for fast cooling. It should be mentioned that all the annealing processes are done in a vacuum environment.

### Characterization

Scanning electron microscope (SEM, FEI – Quanta 200 FEG) operated at 10 kV is used for top and cross sectional imaging. For the optical characterization of the stacks, the normal reflection measurements for the wavelength range of 600 nm to 2000 nm were carried out using Fourier Transform infrared spectroscopy (FTIR, Bruker) and for the remaining part of the visible spectrum, we used a homemade reflection measurement setup in which a Halogen illuminator is connected to a microscope and directed perpendicular to a sample. The reflected light from the microscope was fed to a Newport OSM2 spectrometer and the data was collected by interfacing the spectrometer with PC. The angle resolved reflection characterization was carried out using J.A. Woollam Co. Inc. VASE ellipsometer for different angles of incidence and polarizations.

## Results and Discussion

**Figure 1:**
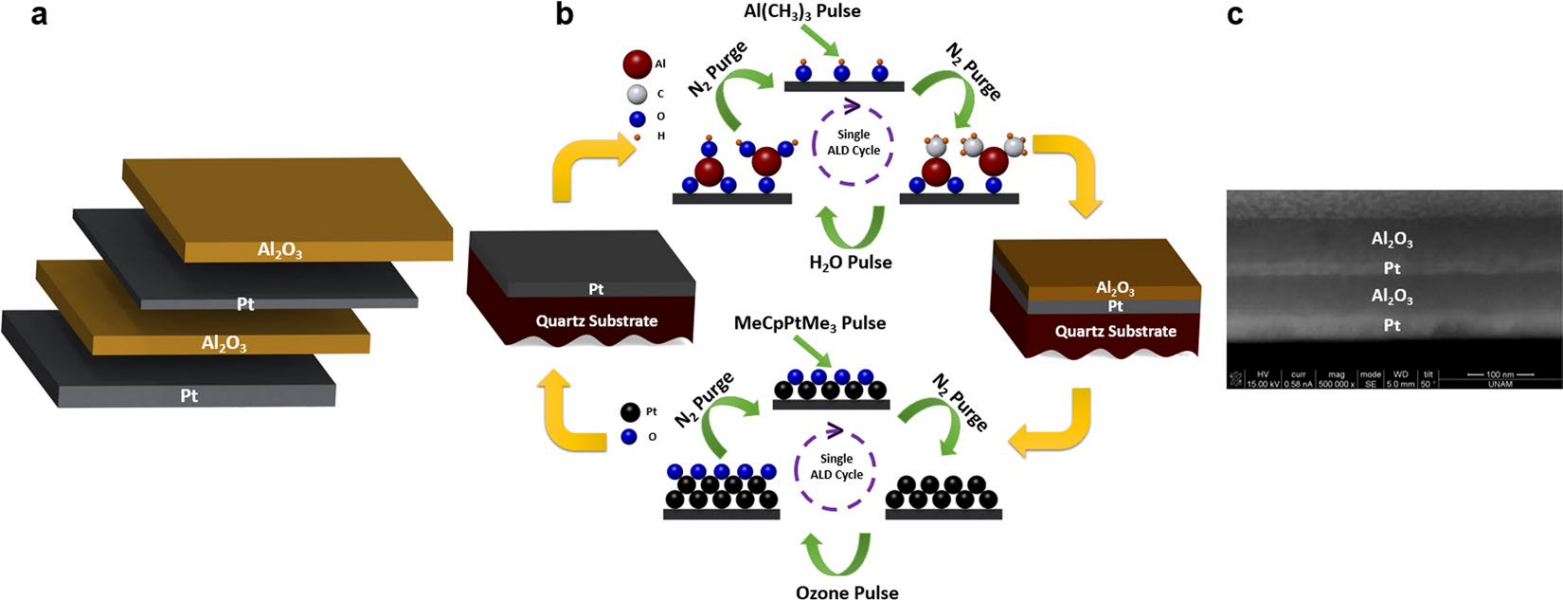
Schematic illustration of (**a**) multilayer absorber design, (**b**) its preparation route in ALD process and (**c**) a cross sectional view from the fabricated design showing the successful formation of multilayer films.

Figure 1 illustrates the schematic of the proposed multilayer design and its preparation route. The structure contains 4 layers that are deposited on a quartz substrate. The bottom layer is a thick (50 nm) platinum (Pt) coating that acts as an ideal mirror reflecting the light back into the cavity. The cavity is a symmetric design of an insulator-metal-insulator stack where the thickness of both Al_2_O_3_ insulator layers is identical and a thin Pt metal layer is sandwiched in between. All the layers are deposited using a single ALD step. A quartz sample is cleaned using a standard cleaning process with acetone, isopropanol, and DI water. Afterwards, the substrate is coated with Pt and Al_2_O_3_ layers by an ALD reactor (Cambridge Nanotech Savannah S100). The substrate temperature is kept at 250 °C during the coating process. Al(CH_3_)_3_ and MeCpPtMe_3_ are taken as precursors for Al_2_O_3_ and Pt deposition, respectively. These precursors together with distilled water are consecutively pulsed and purged into the chamber. The pulse and purge time for Al_2_O_3_ coating is 0.015 s and 10 s, while these amounts for Pt are 0.1 s and 10 s, respectively. The deposition of the multilayer stack was carried out using different numbers of successive cycles with an estimated growth rate of 1.08 Å per cycle for Al_2_O_3_ and 0.9 Å per cycle for Pt layer. By utilizing this architecture, we attempt to design a broadband perfect absorber. To achieve this goal, the optimal performance of the stack is investigated using electromagnetic (EM) full wave Finite Difference Time Domain (FDTD) method using commercial Lumerical software^30^. In the simulation setup, the absorber stack unit cell is excited with a normally incident plane wave in our desired wavelength range and polarization. Considering the fact that the bottom mirror thickness is larger than the skin depth of the light, the transmittance through the multilayer is assumed to be zero (This has been double-checked by simulations). Therefore, the absorption can be simply calculated using the following equation

1 $$A=1\,-\,T\,-\,R=1\,-\,R$$

where *R* is the reflectance off the surface.

**Figure 2:**
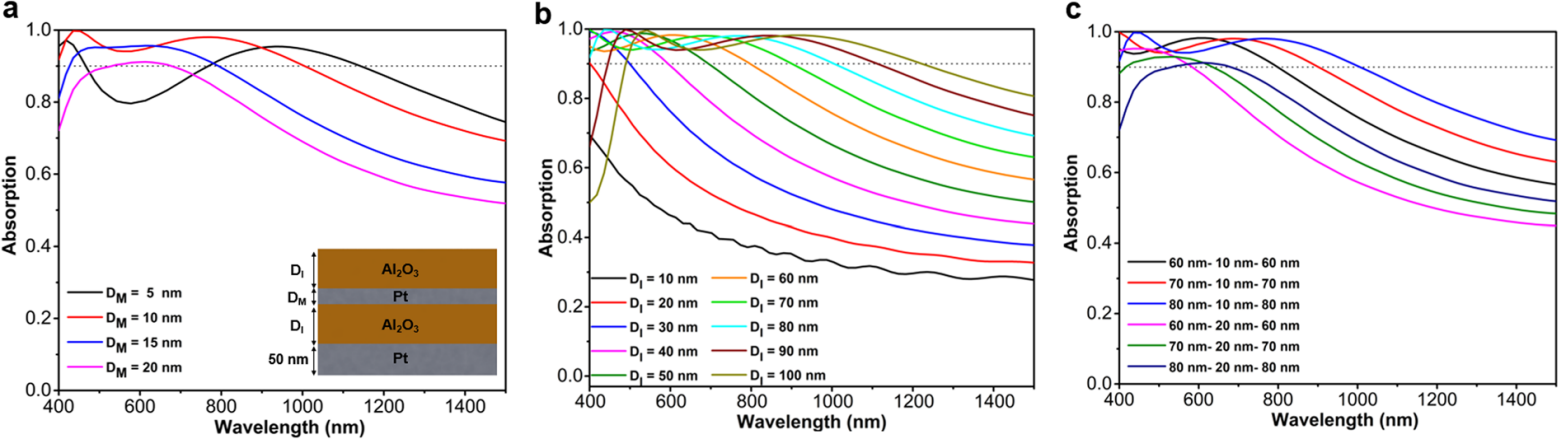
The absorption spectra as a function of incident light wavelength for various (**a**) top Pt layer thickness (D_M_), (**b**) insulator layer thickness (D_I_) and (**c**) six chosen D_I_-D_M_-D_I_ configurations.

In the first step, the impact of both insulator and metal layers on the overall absorption is investigated. Figure 2a illustrates the absorption graph for four different metal layer thicknesses of 5 nm, 10 nm, 15 nm, and 20 nm. As it can be clearly seen, the structure depicts its optimum behavior for a 10 nm thick metal case. Although the thinner layer has a longer absorption tail, it suffers from weak absorption in lower wavelengths. On the other side, thick layers will hamper light penetration with sufficient amplitude to be couple efficiently to the cavity resonator. Therefore, 10 nm case is an optimum amount that retains a reasonable trade-off between light reflection and cavity modes strength. Fixing the Pt metal layer thickness at 10 nm, in the next step, the insulator layer thickness is swept from 10 nm to 100 nm. As the obtained results imply, moving toward a thicker Al_2_O_3_ layer, the absorption tail extends toward the NIR regime. However, similar to the metal layer thickness dependence, thicker Al_2_O_3_ layers lose their absorption capability at lower wavelengths where an abrupt reduction is observed. Considering an absorption above 0.9 as the threshold for bandwidth (BW) determination, the case of 80-10-80 (throughout the paper, we will use “top insulator thickness-metal thickness-bottom insulator thickness” notation to explain the different configurations where the numbers have nanometer unit) has the optimum absorption with a BW of 600 nm (from 400 nm to 1000 nm) that covers the whole visible spectrum. The response of the design with different metal and insulator thicknesses has been further compared in Fig. 2c. This comparison also confirms the conclusions that we derived in the previous parts. To have a comprehensive comparison, a contour plot for the absorption spectra of the design as a function of metal layer thickness (D_M_) and insulator layer thickness (D_I_) is depicted in Supplementary information (Fig. S1).

**Figure 3:**
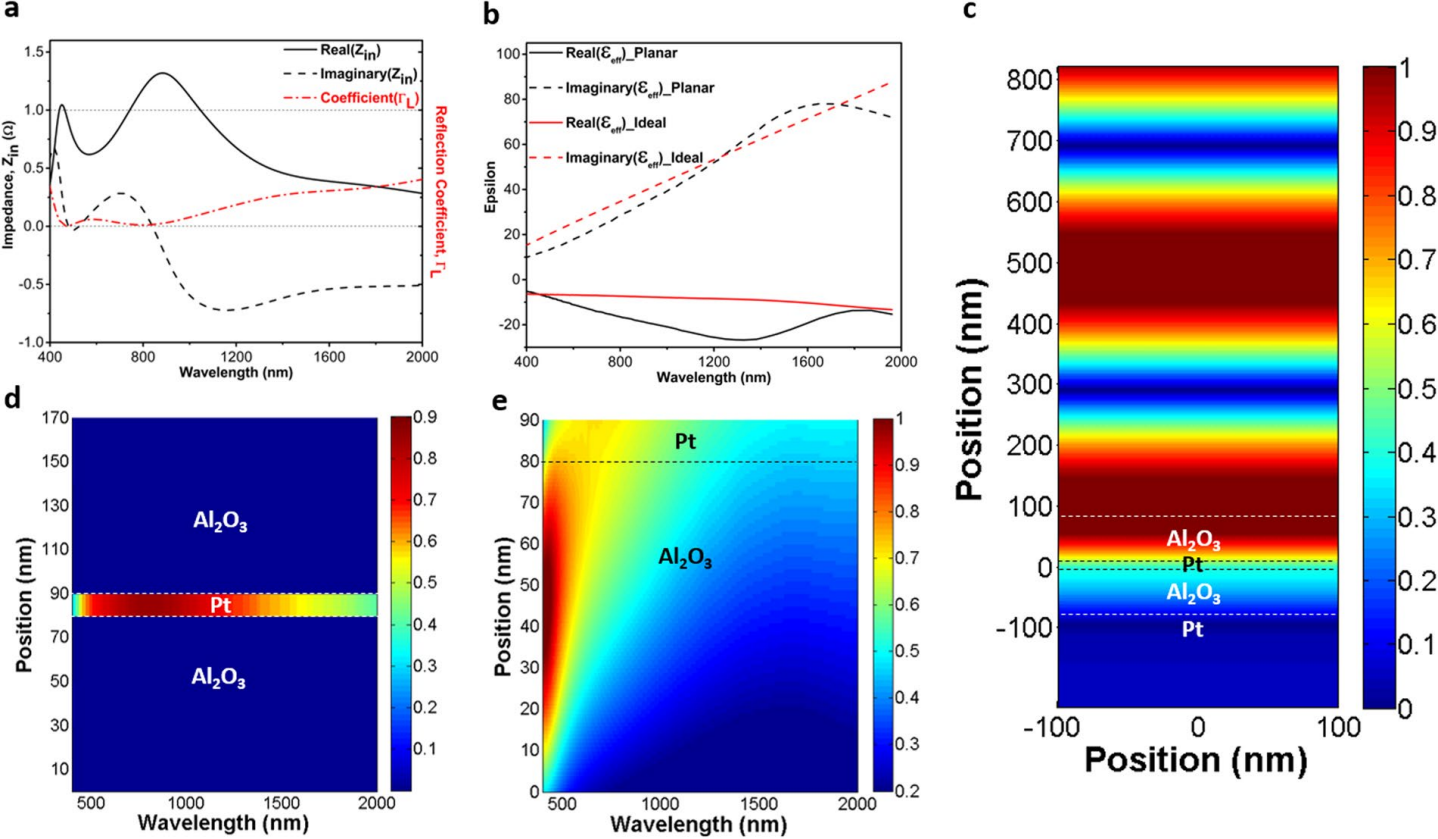
(**a**) Calculated impedance and reflection coefficient, (**b**) epsilon of the ideal metal and Pt (**c**) mode profile for electric field distribution (E_x_) of the incident light from air into the cavity design, and simulated contour plots of (**d**) total absorber power and (**e**) electric field profile as a function of wavelength and position in the cavity structure for the 80-10-80 configuration.

**Figure 4:**
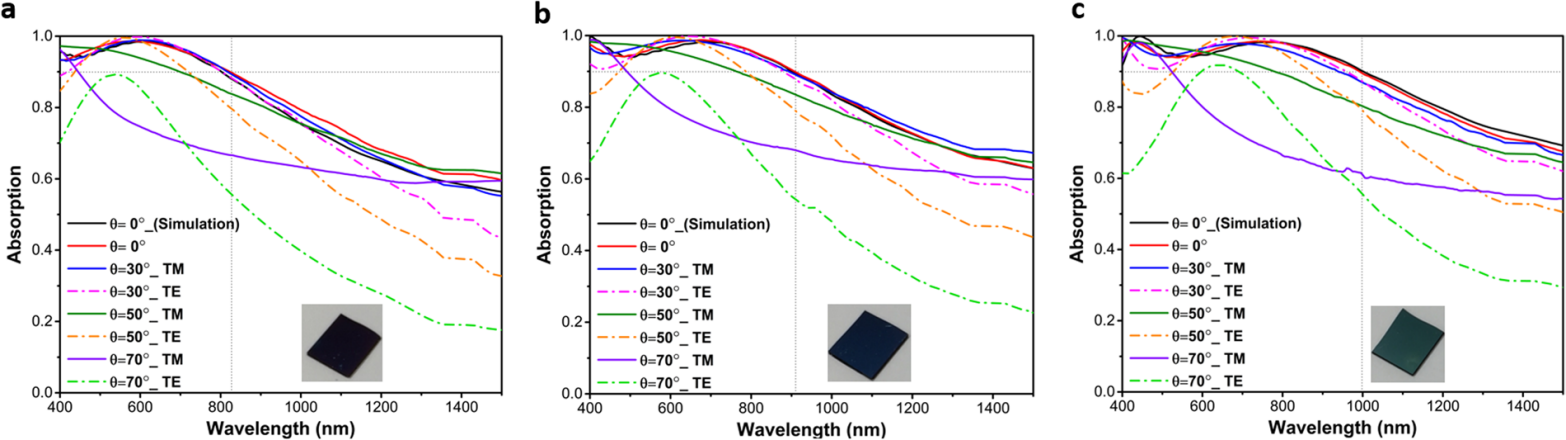
Measured absorption spectra of fabricated planar multilayer stack in different incidence angles of 0°, 30°, 50°, and 70° with both TE and TM polarizations for (**a**) 60-10-60, (**b**) 70-10-70 and (**c**) 80-10-80 configurations.

**Figure 5:**
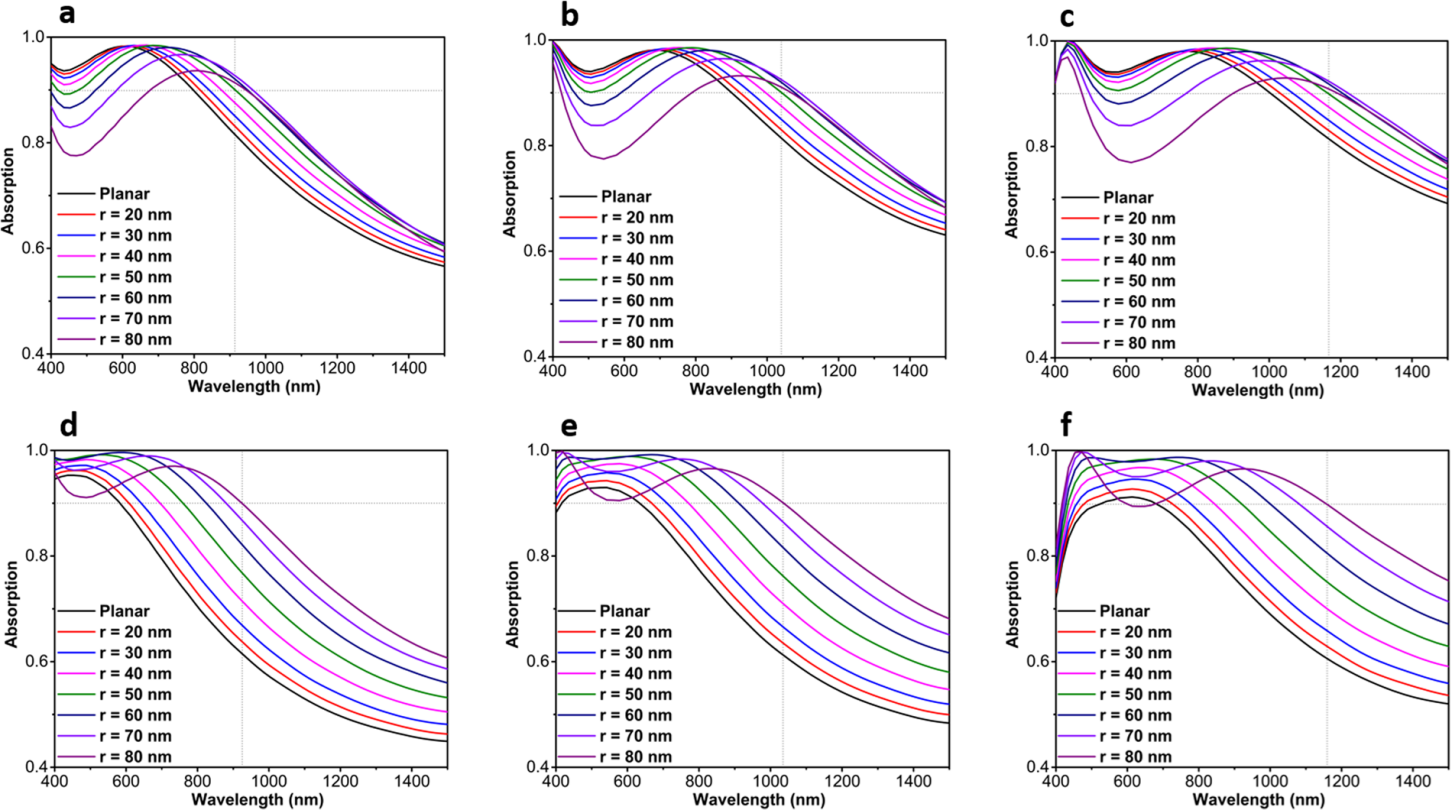
Simulated absorption response of the MIMI stack with nanoholes plasmonic layer with different radius values (from 20 nm to 80 nm) for different configurations of (**a**) 60-10-60, (**b**) 70-10-70 and (**c**) 80-10-80, (**d**) 60-20-60, (**e**) 70-20-70 and (**f**) 80-20-80.

**Figure 6:**
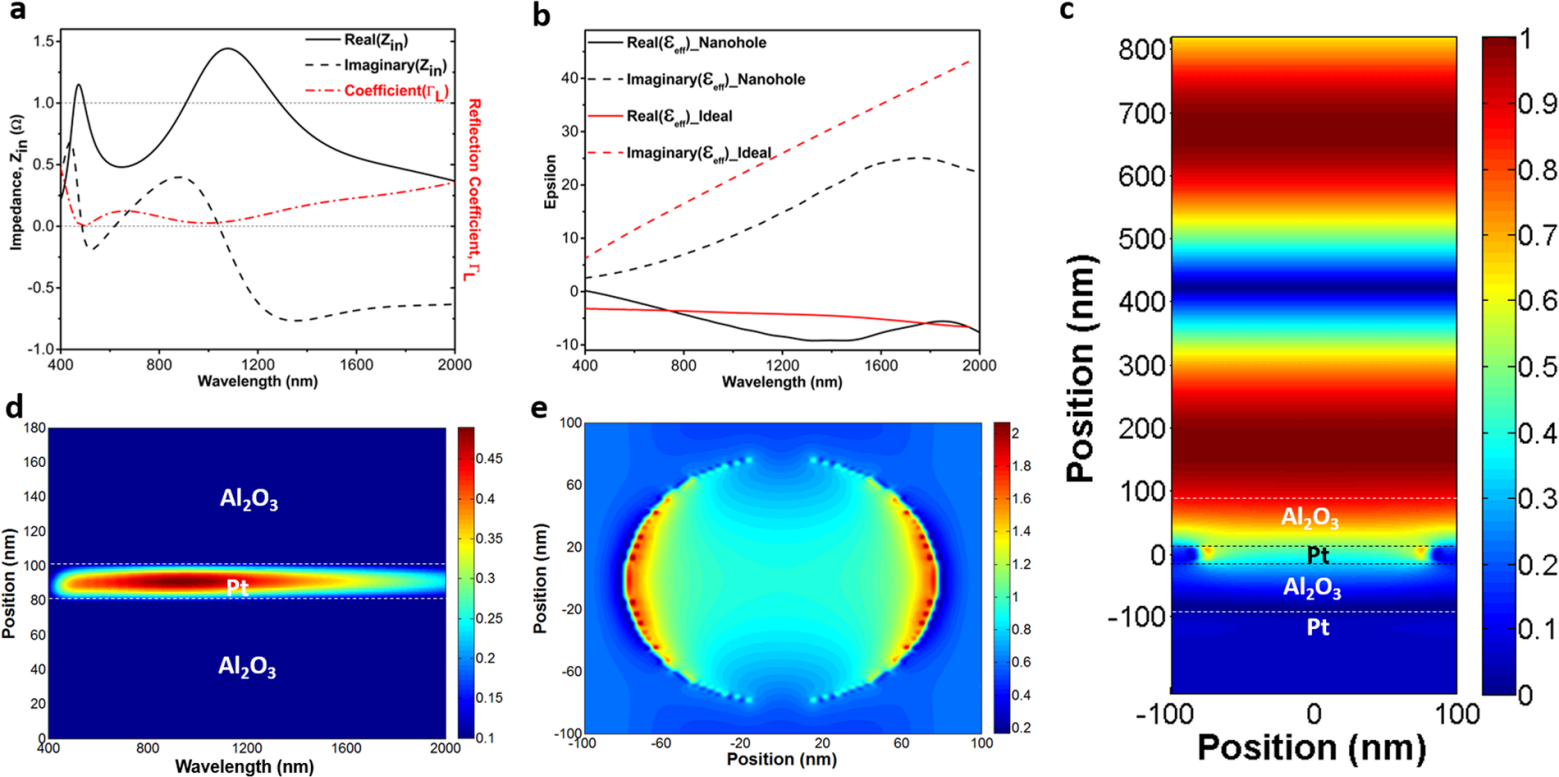
(**a**) Calculated impedance and reflection coefficient, (**b**) epsilon of the ideal metal and nanoholes patterned Pt (**c**) mode profile for electric field distribution (E_x_) of the incident light from air into the cavity design, and simulated contour plots of (**d**) total absorber power and (**e**) electric field intensity in the middle of Pt layer that have dipole like field distribution, as a function of wavelength and position for 80-20-80 nanoholes structure with a hole radius of 80 nm.

**Figure 7:**
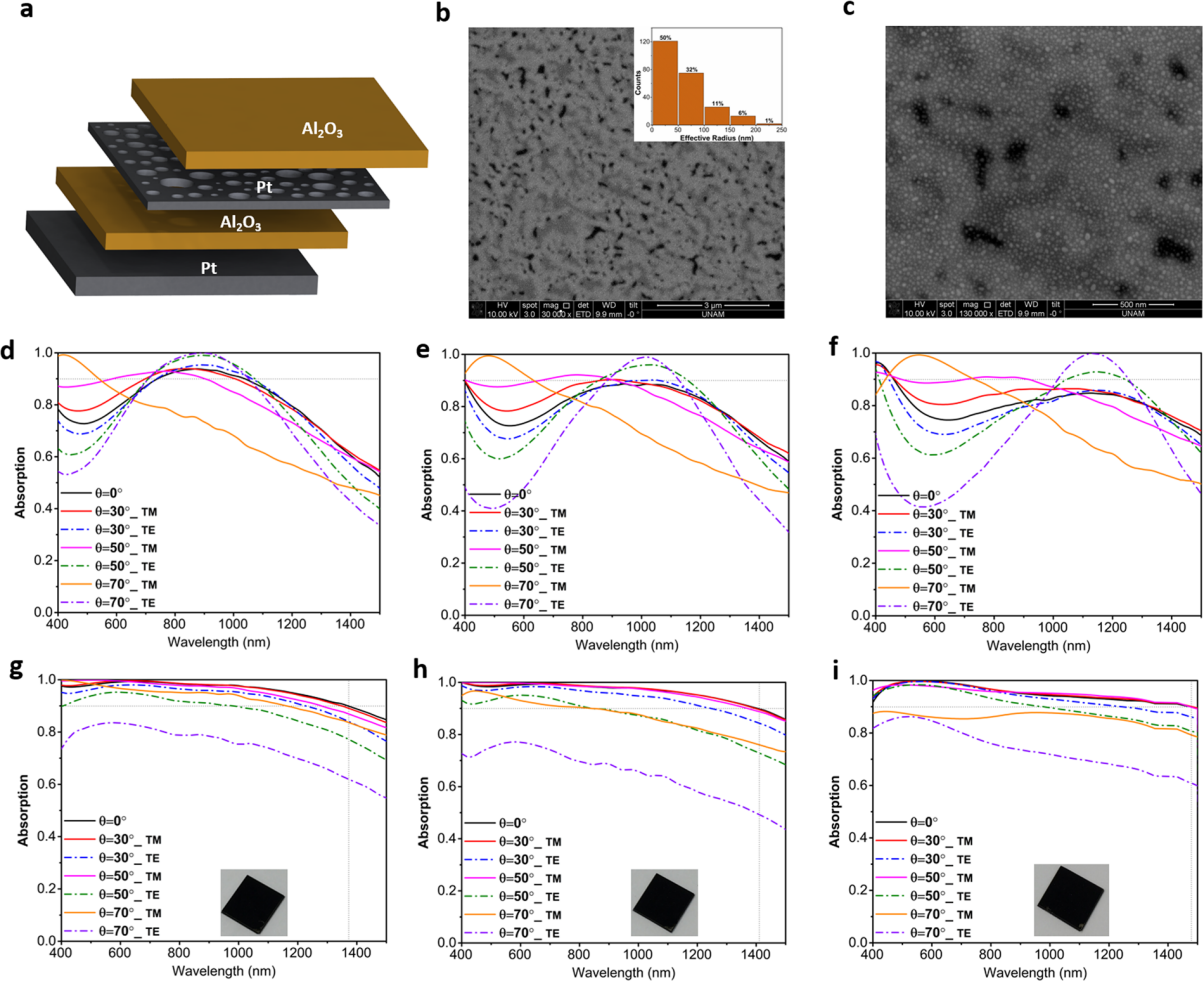
(**a**) Our desired multilayer stack design and (**b**,**c**) SEM image of the fabricated film with widely size distributed nanoholes formed on the Pt layer. The absorption spectra of the nanoholes based design for different angles of incidence and different polarization in various stack geometries of (**d**) 60-10-60, (**e**) 70-10-70, (**f**) 80-10-80, (**g**) 60-20-60, (**h**) 70-20-70 and (**i**) 80-20-80.

To have a better understanding on the origin of this ultra-broadband light absorption in the MIMI design, a theoretical modeling approach is adopted to explain this behavior. As theoretically demonstrated^24^ and experimentally verified by^26^, if the impedance-matched condition (Z = 1Ω) is met for a planar MIMI multilayer in which the metal layer is ultra-thin, the system can act as a broadband perfect absorber. Moreover, in this condition, it is possible to describe the real and imaginary parts of the permittivity of the ideal thin metal layer as^26^:

2 $$\varepsilon {^{\prime} }_{ideal}(\omega )=\frac{-{\varepsilon }_{A{l}_{2}{o}_{3}C}}{n^{\prime\prime} {m}^{\omega dpt}},\varepsilon ^{\prime} {^{\prime} }_{ideal}(\omega )={\varepsilon }_{A{l}_{2}{o}_{3}}\frac{(n^{\prime\prime} {m}^{C-\varepsilon }A{l}_{2}{o}_{3}\omega dpt)}{n^{\prime\prime} m\omega dpt}$$

where $n{^{\prime\prime} }_{m}$ is the imaginary part of the refractive index of the metallic reflector, placed beneath the insulator-metal-insulator (IMI) structure. Therefore, a metal that has real and imaginary permittivity close to these ideal values can provide an ultra-broadband light absorption. To understand how well Pt impedance is matched to this data, we have calculated the input impedance of our multilayer design using the well-known effective parameter extraction method^31^. Moreover, in order to obtain better insight into the mechanism of light reflection from the surface, reflection coefficient $({\Gamma }_{L}=\frac{{Z}_{in}-1}{{Z}_{in}+1})$ is also calculated using a simple transmission theory assumption. As it can be clearly seen in Fig. 3(a), real values of the input impedance of the planar structure fluctuates around Z = 1Ω and reaches 1 and 1.2 at λ = 470 nm and λ = 800 nm, respectively. In addition, at the mentioned wavelengths, the reflection coefficient (Γ_L_) reaches its minimum values. This point evidently emphasizes how almost-zero reflections (perfect absorptions) are connected with the impedance-matched condition of the structure. Therefore, since the impedance-matched condition is satisfied, it is justifiable to calculate the ideal permittivity values of the metal layer using Eq. (2) and compare them with the corresponding ones of Platinum^32^. This comparison is illustrated in Fig. 3(b). As shown in this figure, there is a fare match between the real (solid curves) and imaginary (dashed curves) values of ε_Ideal_ and ε_Pt_. This agreement demonstrates that in order to achieve a perfect broadband absorber, Pt is as excellent as Cr^26^ for the middle layer of the MIMI absorber. In support of the aforementioned points, Fig. 3(c) illustrates how an incident plane wave at λ = 800 nm, which is illuminated from air (z > 850 nm) to the planar Pt- Al_2_O_3_-Pt-Al_2_O_3_ structure, penetrates into the stack without any change in its phase and is finally trapped and absorbed by the cavity. Light absorption and electric field intensity distributions over the multilayer design, as a function of incident light wavelength, are studied as shown in Fig. 3(d–e). Figure 3d demonstrates that light absorption is dominant in the middle Pt layer and is very weak in the Alumina insulators. This can be attributed to the high extinction coefficient of the Pt metal layer. Figure 3e also sketches the E-field distribution over the bottom MIM design. First feature that is bolded in this contour plot is the low E-field intensity in the Pt metal layer. This weak intensity distribution confirms a low-quality factor of the resonator (which is expected for wideband resonance based absorptions). Moreover, E-field profile distributions over the cross-section could illustrate the reason for the broad light absorption capability of the design. Light penetration into the MIM cavity gets weaker when the incident light wavelength increases and this in turn explains why the cavity modes strength gets diminished at longer wavelengths. To experimentally validate our findings, we have fabricated 3 different configurations of 60-10-60, 70-10-70, 80-10-80 using the single ALD step. Figure 4(a–c) depicts the obtained absorption amounts for normal and different angles incident light for both TM and TE polarizations. As shown in this figure, the experimental results and their simulation counterparts for normal incident cases are in good agreement for all three cases that shows the accuracy of our findings in simulation and modeling parts. The absorption curve retains values above 0.9 up to 825 nm, 910 nm, and 1000 nm for the cases of 60-10-60, 70-10-70, 80-10-80, respectively. Therefore, the maximum bandwidth achievable with a simple planar structure is 600 nm (from 400 nm to 1000 nm). The angular dependence of absorption spectra for TE and TM polarizations should also be considered to have a better qualitative comparison. For incidence angles of $\theta  < 50^\circ$, the BW is almost unaltered. For $\theta =50^\circ$ incident light angle, the absorption is still above 80 percent within the BW for all three cases. However, for larger angles, the high cavity absorption response is no longer sustained. As explained in previous parts, the proposed metal-insulator multilayer is essentially a low quality-factor asymmetric Fabry-Perot design. In this planar stack, every layer has a function to ensure a broad light absorption. In a general view, the top insulator layer acts as an antireflective coating that provides a gradual impedance matching between an air and underlying metal-insulator-metal cavity. Therefore, the main mechanism for light absorption in such a structure is the cancellation of reflection from different metal-insulator interfaces. This means that an ultra-broadband response requires a perfect impedance matching between air and MIMI stack in a broad frequency range and this is restricted with the inherent optical properties of the metal and insulator layers. A substantial BW improvement can be accomplished utilizing plasmonic designs at the embedded Pt layer where light can be confined in sub-wavelength units. This design should provide sub wavelength light confinement but should not hamper partial light reflection/transmission characteristic of the layer. Satisfying these conditions can further increase the light absorption edge and its strength. Nanoholes as plasmonic units that can confine light in sub-wavelength scales and, at the meantime, do not demolish layer continuity are a proper option. Figure 5 depicts a comparison among planar and different-sized nanoholes absorptions for different IMI configurations. In the case of nanohole design, simulations are carried out in a square unit cell with a periodicity of P = 200 nm. Taking the Pt layer thickness as 10 nm (Fig. 5a–c) and 20 nm (Fig. 5d–f), we performed simulations for nanoholes in a radius (r) range of 20 nm–80 nm. As the findings illustrate, for all the cases, the nanohole design reveals superior absorption compared to the planar one. The introduction of nanoplasmonic unit into the middle metal layer has extended the absorption edge toward longer wavelengths. However, in the case of thin Pt layer (10 nm), for radii above 50 nm, a valley starts to grow at lower wavelengths and deteriorates the overall response. However for 20 nm thick Pt layer, the absorption stays above 0.9 up to nanoholes sizes of *r* = 80 *nm* and generally absorption capacity improvement is more pronounced in the thicker layer (D_M_ = 20 nm). This response can be justified by considering the ultrathin thickness of metal layer. In a MIM cavity configuration, to strengthen cavity induced modes, the top layer thickness should be thick enough so that light can efficiently bounce back and forth between two metal-insulator interfaces in the cavity. This layer should not be too thick to let the reflection off the surface dominate the transmission. Our theoretical calculations are also in line with our results. Following the results shown in Fig. 3, the effective impedance and reflection coefficient of the nanohole Pt-Al_2_O_3_-Pt-Al_2_O_3_ absorber is illustrated in panel (a) of Fig. 6. As explained earlier, for the nanoholes absorber, the middle 20 nm-thick Pt layer is patterned with holes of a radius 80 nm in a square lattice with a period of 200 nm. It is observed from this figure that by patterning the middle Pt layer it is possible to considerably red-shift the impedance-matched bandwidth of the absorber wavelengths; these wavelengths are shifted from 470 nm and 800 nm in the unpatterned case to 490 nm and 1000 nm in the patterned case, respectively. Therefore, the upper impedance-matched wavelength (λ = 1000 nm) is considerably transformed inside the near-IR region. Consequently, we are able to noticeably broaden the perfect absorption. It is noteworthy that our numerical calculations show that, by using the same method, it is possible to increase the absorption bandwidth of the Cr-Al_2_O_3_-Cr-Al_2_O_3_ system up to 1390 nm (results are not described here). Moreover, we have also extracted the effective permittivity of the patterned 20 nm-thick Pt layer and compared it with the ideal permittivity of the middle metal layer obtained by Eq. (2). It is clear from Fig. 6b that the real part of permittivity of the patterned Pt layer (black-solid curve) has closer values to those of the ideal ones (red-solid curve) compared with the ones illustrated in Fig. 3b. This better matching, in fact, leads to the broader impedance-matching for the overall structure. On the other hand, as expected, from the dashed-black curve in Fig. 6b we understand that patterning the Pt thin film causes the reduction of the imaginary values of the permittivity in comparison to those of ε_Pt_″ as represented in Fig. 3c. Complementing our explanations on how the impedance-matched condition affects the absorption of the system, comparing Fig. 3c with Fig. 6c, we represent the mode profile at λ = 1000 nm. This figure clearly illustrates that light with zero-change in its phase penetrates inside the absorber and is quickly blocked by it due to the support of localized surface plasmon polaritons (LSPPs) by the system; notice how LSPPs changes the mode profile at Z = 0 in Fig. 6c in comparison with the one represented in Fig. 3c. This plot shows that light is more strongly confined at the hot spots of nanoholes and its penetration depth into the bottom insulator layer is much less compared to planar design. Similar to planar design, for all frequencies, the main absorption is concentrated at the Pt layer, as shown in Fig. 6c. The electric field (E_x_) distribution in the mid plane of Pt layer shows that the absorption is due to the existence of an electric dipole resonator where this dipole captures light in sub-wavelength units and hampers its penetration. Taking all of the afore-mentioned discussions into consideration, it should be noted that up to certain point, the absorption edge is stretched out moving toward larger nanoholes while the amount is above 0.9. After an optimum value, a broad dip begins to grow and diminishes the multilayer absorption capacity. This reduction is much more strong for D_M_ = 10 nm cases. The absorption is above 0.9 for all radii but it experiences a broad dip reaching an amount below 0.8 over the entire visible frequencies. On the other end of the scale, however, small nanoholes have much stronger absorption in lower wavelengths. Therefore, an optimum design should be a combination of different sized nanoholes where large holes are responsible for an absorption tail extension in high wavelengths and small ones will ensure flat absorption in lower λs. These design requirements should be fulfilled using a large scale compatible fabrication method. One of the methods that can be used to get such nanostructures is de-wetting. In the de-wetting process, the metal layer is annealed in high temperatures for an optimized time duration to obtain our desired nano pattern. Based on the annealing duration and its temperature, we can get nanoholes, nanodots or some other nano network structures. Generally, upon exposing the metal layer to a temperature around melting point, the film starts to melt and recrystallize. During this recrystallization, nanoholes start to appear and expand until these nanoholes overlap and turn to nanoislands (or nanodots). Therefore, to obtain nanoholes, we need to keep the annealing time short enough. Annealing process is carried out in a homemade tube-furnace under vacuum conditions. To obtain nanoholes from Pt, film temperature is raised up to 800 °C with a rate of 30 °C/min, kept at this amount for our desired time duration (5 min in 10 nm thick Pt and 12 min for 20 nm one), and abruptly exposed to room temperature. Figure 7a schematically illustrates our targeted design structure. The SEM images at Fig. 7b,c depict the top layer morphology of de-wetted Pt film. As this image clearly displays, disordered nanoholes with wide radius ranges are formed in a 20 nm thick Pt film. The effective radius ($\sqrt{(hole\,area)/\pi }$)) size distribution of these nanoholes is also provided in the inset of the figure. We fabricated an MIMI stack with a different insulator (D_I_ = 60 nm, 70 nm and 80 nm) and metal thicknesses (D_M_ = 10 nm and 20 nm) based on this nanostructure design. As Fig. 7d–f present, the absorption responses for 10 nm thick Pt layer (60-10-60, 70-10-70, 80-10-80) do not retain its high absorption capability at a broad frequency range. The absorption experiences a broad and deep minimum at the low values of the wavelength and this groove gets a slight red shift when we move to thicker insulator layers. This finding is in agreement with our previous results. In Fig. 5a–c, we showed that for radii above 50 nm, the absorption of the MIMI stack experiences a reduction in low wavelengths and this dip moves to higher lambdas for a thicker Al_2_O_3_ layer. Moreover, in the inset of Fig. 7b, we can see that more than 50 percent of our holes have a radius above 50 nm. In addition, the trend for different insulator thicknesses is also in agreement with our simulations. Angular dependence of the absorption for TE and TM polarizations has been studied in this figure as well. For TM polarizations, the absorption capability of the system has been even improved for incident angles up to $\theta =50^\circ$, especially at lower wavelengths. However, the same behavior is not repeated by TE polarization. This can be elucidated better by taking surface plasmon polariton (SPP) propagation in a metal-insulator interface into account. When light comes with an angle, SPP modes start to propagate in the MI interface and will be coupled to localized SPP at the positions that we have nanoholes. However, these modes cannot be supported for TE polarized incident light. In addition to this, at large angles of incidence, the path length of light propagation in the nanostructures increases and, therefore, light is absorbed stronger in the plasmonic structure at higher angles of incidence. Unlike this case, 20 nm thick nanoholes demonstrate a flat, ultra broadband and wide angle absorption where the absorption edge (considering a threshold of 0.9) is extended up to 1490 nm and a BW of ~1100 nm can be attained for 80-20-80 configuration. The stack keeps its high response even for 70° incidence angles where the absorption is above 0.8 from the entire wavelength range for TM polarized light, and above 0.7 for TE polarization. A similar behavior is recorded with 70-20-70 and 80-20-80 cases. This can stem from the fact that nanoholes have wide radius distributions which ensure light absorption in a wide frequency range. This is also in agreement with our simulation results that show a quasi-flat absorption for the nanoholes radius amounts of approximately 80 nm. The results obtained in this study are also extendable to other types of MI pairs based absorbers where a simple, large scale compatible annealing step can create nano plasmonic patterns in the middle metal layer and this in turn would improve light absorption strength and bandwidth.

## Conclusion

In summary, an ultrathin MIMI multilayer structure for ultra-broadband and wide angle perfect absorption was designed, fabricated, and characterized. The fabrication route of this multilayer stack was lithography free and the use of ALD as a chemical vapor deposition method increases the fabrication reliability. We demonstrated that the introduction of disordered nanoholes can significantly improve the light absorption BW and its omnidirectionality. In the best case, we were able to obtain a perfect absorber with absorption above 0.9 throughout the entire wavelength range of 400 nm–1490 nm. The findings and proposed fabrication steps can develop an efficient approach to design other types of multilayer stacks where our simple route can result in significant performance enhancement.

## Electronic supplementary material

SI
