# Supplementary material for: Disordered Nanohole Patterns in Metal-Insulator Multilayer for Ultra-broadband Light Absorption: Atomic Layer Deposition for Lithography Free Highly repeatable Large Scale Multilayer Growth
Source: Sci Rep. 2017 Nov 8;7:15079. doi: 10.1038/s41598-017-15312-w (PMC5678139; doi:10.1038/s41598-017-15312-w)
Supplement: Supplementary file 1 — SI [file 41598_2017_15312_MOESM1_ESM.pdf]

Supporting Information

Disordered Nanohole Patterns in Metal-Insulator Multilayer for Ultra-broadband Light Absorption: Atomic Layer Deposition for Lithography Free Highly repeatable Large Scale Multilayer Growth

Amir Ghobadi[1, 2, *], Hodjat Hajian[1], Sina Abedini Dereshgi[1, 2], Berkay Bozok[1, 2], Bayram Butun[1], Ekmel Ozbay[1, 2, 3, 4, *]

[1]NANOTAM-Nanotechnology Research Center, Bilkent University, 06800 Ankara, Turkey

[2]Department of Electrical and Electronics Engineering, Bilkent University, 06800 Ankara, Turkey

[3]Department of Physics, Bilkent University, 06800 Ankara, Turkey

[4]UNAM-Institute of Materials Science and Nanotechnology, Bilkent University, Ankara, Turkey

* Corresponding authors: amir@ee.bilkent.edu.tr, ozbay@bilkent.edu.tr

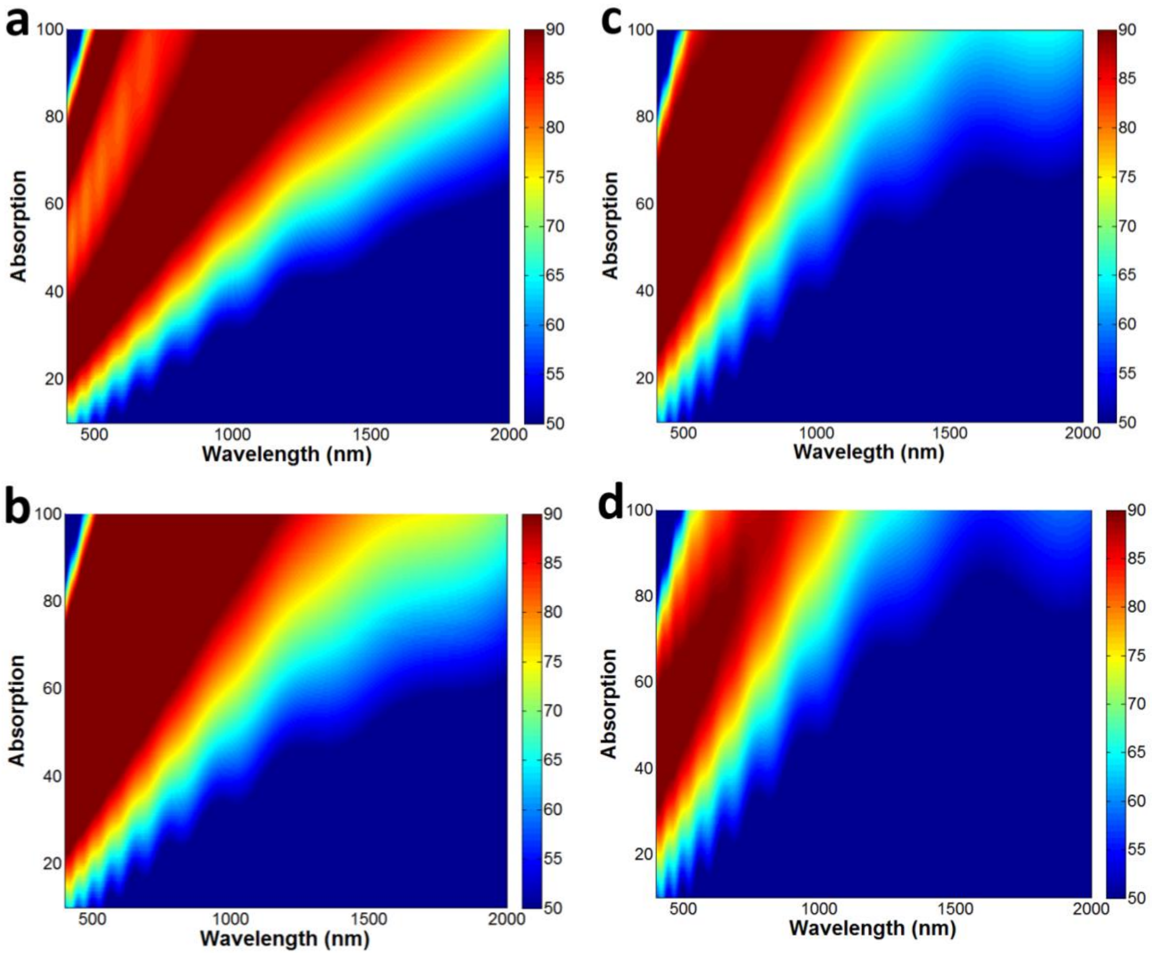

Figure S1. The absorption contour plot as a function of incident light wavelength and insulator layer thickness for different metal thicknesses of a) D_M=5 nm, b) D_M=10 nm and c) D_M=15 nm, and d) D_M=20 nm.
